# Rumen fermentation and microbiota in Shami goats fed on condensed tannins or herbal mixture

**DOI:** 10.1186/s12917-024-03887-2

**Published:** 2024-01-31

**Authors:** Alaa Emara Rabee, Moustafa Mohamed M. Ghandour, Ahmed Sallam, Eman A. Elwakeel, Rasha S. Mohammed, Ebrahim A. Sabra, Adel M. Abdel-Wahed, Disouky Mohamed Mourad, Amal Amin Hamed, Osama Raef Hafez

**Affiliations:** 1 Animal and Poultry Nutrition Department, Desert Research Center, Ministry of Agriculture and Land Reclamation, Cairo, Egypt; 2 Animal and Poultry Breeding Department, Desert Research Center, Cairo, Egypt; 3 Department of Animal and Fish production, Faculty of Agriculture, Alexandria University, Alexandria, Egypt; 4 Animal and Poultry Health Department, Desert Research Center, Ministry of Agriculture and Land Reclamation, Cairo, Egypt; 5 Genetic Engineering and Biotechnology Research Institute, University of Sadat City, Sadat City, Egypt; 6 Botany and Microbiology Department, Faculty of science, Cairo University, Cairo, Egypt

**Keywords:** Goats, Phytochemicals, Tannins, Herbal mixtures, Rumen fermentation, Bacteria, Archaea

## Abstract

**Background:**

Phytochemical compounds can modify the rumen microbiome and improve rumen fermentation. This study evaluated the impact of supplementation with tannin and an herbal mixture containing ginger (*Zingiber officinale*), garlic (*Allium sativum*), Artemisia (*Artemisia vulgaris*), and turmeric (*Curcuma longa*) on the rumen fermentation and microbiota, and histology of rumen tissue of goats. Eighteen Shami male goats were divided into three groups (*n* = 6): non-supplemented animals fed the basal diet (C, control); animals fed basal diet and supplemented with condensed tannin (T); and animals fed basal diet and supplemented with herbal mixture (HM). Each animal received a basal diet composed of Alfalfa hay and a concentrate feed mixture.

**Results:**

Group HM revealed higher (*P* < 0.05) rumen pH, total volatile fatty acids (VFA), acetic, propionic, isobutyric, butyric, isovaleric, and valeric. Principal Co-ordinate analysis (PCoA) showed that rumen microbial communities in the control group and supplemented groups were distinct. The supplementation increased (*P* < 0.05) the relative abundances of phylum Bacteroidota and Proteobacteria and declined (*P* < 0.05) Firmicutes and Fibrobacterota. Additionally, the dominant genus *Prevotella* and *Rikenellaceae RC9* gut group were increased (*P* < 0.05) and the family Ruminococcaceae was declined (*P* < 0.05) due to the supplementation. The supplementation decreased (*P* < 0.05) the archaeal genus *Methanobrevibacter* and increased (*P* < 0.05) *Candidatus Methanomethylophilus*. Tannin supplementation in T group shortened the rumen papillae.

**Conclusions:**

The results revealed that the herbal mixture might be used to alter the rumen microbiota to improve rumen fermentation.

## Background

The digestion of plant biomass in the rumen relies on interactions between a complex assemblage of bacteria, archaea, fungi, and protozoa [1–4]. Rumen bacteria predominate the rumen microbiome and they ferment a wide variety of dietary components such as cellulose, hemicellulose, starch, pectin, and protein. VFA and microbial protein are the main products of rumen fermentation and they provide the host animal with most of energy and protein requirements. Additionally, rumen archaea uses fermentation gases such as hydrogen (H_2_) and carbon dioxide (CO_2_) besides methyl group derived from acetic acid, and format to generate methane (CH_4_) [5]. Methane represents a 2–12% loss of the gross energy feed intake of host animal and it is one of the main reasons for climate change [5, 6]. Rumen methanogens interact with H_2_ producers and utilizers; therefore, modifying rumen microbial ecosystem is a main target for studies that aim to improve animal efficiency and decrease methane emission [5, 7]. Dietary intervention is the main driver of changes in the rumen microbiome. Consequently, understanding the modifications of rumen microbiome under different diets or feed additives opens the door to designing suitable strategies to improve animal productivity [6]. Phytogenic feed additives are emerging additives to enhance animal’s productivity through the modification of the rumen ecosystem [8, 9]. These additives have been used in the form of extracts, herbal mixtures, or oils.

Herbal plants that are rich in phytochemicals, including ginger (*Zingiber officinale*), garlic (*Allium sativum*), artemisia (*Artemisia vulgaris*), and turmeric (*Curcuma longa*) [10–14]. Ginger contains several substances such as gingerol and gingerdione [10], and garlic contains several organosulfur compounds, including allicin, diallyl sulfide, γ-glutamylcysteine, and S-allyl cysteine [11, 12]. Furthermore, artemisia contains artemisinin [13], and turmeric contains curcumin and turmerones besides several other substances and oils [14]. Additionally, phytogenic compounds that were commonly observed in ginger, garlic, artemisia, and turmeric, including phenolic compounds, tannins, saponins, and flavonoids [10–14]. Therefore, these plants have been used as growth promoters as well as rumen microbiome modifiers to improve rumen fermentation and promote animal health and performance [10–15]. Phytogenic compounds were supplied to farm animals separately or mixed to generate the synergistic effect of the combination. Williamson [16] reported that synergistic interactions between the bioactive compounds of herbal plants are a vital part of their efficacy and total herbal extracts show a better effect than an equivalent dose of an isolated compound. In addition, herbal plants provide essential amino acids, carbohydrates (sucrose and glucose), vitamins, essential oils, and minerals [17–20]. Several studies applied phytochemical mixtures to modify rumen fermentation in order to improve animal efficiency. Petric et al. [21] noticed that herbal mixture supplementation in lambs affected the bacterial population and the abundance of *Ruminococcus* and *Fibrobacter*. In water buffalo, Hassan et al. [22] reported that phytochemicals mixtures increased the relative abundance of phylum Firmicutes and Proteobacteria, while the relative abundances of Bacteroidetes and Spirochaetes besides genus *Prevotella* were declined. Kholif et al. [8] indicated that herbal mixture increased total VFA and propionic acid besides the relative abundances of propionate-producing bacteria such as family Prevotellaceae and Veillonellaceae, and declined the methane-producing archaea, Methanobacteriaceae. Apart from herbal mixtures, separated phytogenic compounds were used to modify the rumen microbiome. Li et al. [23] noticed that flavonoids supplementation increased rumen acetate, propionate, and total VFAs, and declined the alpha diversity indices in buffalo. Furthermore, flavonoids increased the abundance of Proteobacteria and declined the relative abundances of Actinobacteria, Acidobacteria, Chloroflexi, and Patescibacteria. Salami et al. [24] supplemented lambs with condensed tannins and observed that tannin did not affect rumen fermentation parameters or the bacterial population, while the archaeal population was declined. Moreover, tannin decreased the relative abundances of cellulolytic bacteria, *Fibrobacter*, and methanogen *Methanosphaera*. Hassan et al. [25] indicated that Saponins have a modulating effect on rumen fermentation as they declined the population of protozoa and methanogens; therefore, methane production was declined. Rabee et al. [26] analyzed the bacterial community colonized tannin-rich plants in the rumen of camels and noticed that higher Bacteroidetes was observed in lower-tannin plants and the relative abundances of Firmicutes, Proteobacteria, and Tenericutes were higher in tannin-rich plants. Moreover, *Fibrobacteria* showed sensitivity to tannins and *Prevotella* showed resistance to some types of tannins. However, studies that compared the effect of separated extracted phytochemicals to mixed compounds or herbal mixtures containing different compounds on rumen fermentation and microbiota are not available.

Shami (Damascus) goat is one of the main goat’s breeds in Egypt that is contributing substantially to meat and milk production in Egypt; however, goats’ production in developing countries is challenged by feeding and health problems [27]. Consequently, safe and available feed additives are required to modulate the rumen fermentation and improve goats’ performance and health. Phytochemicals-rich plants are commonly used in feeding ruminant animals in arid regions [26]. However, the effect of these plants separately or combined in different combinations on rumen ecosystem and animal performance, need to be assessed as different action mechanisms are expected due to the variation in the composition and secondary metabolites [28]. On the other hand, comparative studies that aim to compare the effect of separate phytochemicals to herbal plants containing mixtures of phytochemicals are not available. In addition, no available studies on the effects of phytochemicals on rumen fermentation and the microbiome in Shami goats. Therefore this study aims to investigate the effect of condensed tannin and herbal mixture on the performance, rumen fermentation, rumen microbiota, and rumen histology of goats.

## Methods

### Ethics

The study, including euthanasia of the experimental animals was conducted under the guidelines of the Animal Care and Use Committee in the Department of Animal and Poultry Production, Desert Research Center, Egypt (Project ID: 43,213). Alexandria University Research Ethics Review Committee, Faculty of Agriculture, University of Alexandria, Egypt approved all experimental procedures (Reference: Alex. Agri. 082305306). All methods and protocols in this study are in compliance with the ARRIVE guidelines.

### Animals and diets

This experiment was carried out at Maryout Research Station, Desert Research Center, Alexandria, Egypt. Eighteen male Shami goats (26.66 ± 1.31 kg initial body weight and 11–12 months of age) were selected randomly for this 90-day experiment and divided into three treatments, six animals per treatment. The goats used in this study were the progeny of the goats’ herd of Maryout Research Station, Desert Research Center, Alexandria, Egypt. The three groups received the same basal diet with a 70% concentrates mixture and 30% Alfalfa hay (*Medicago sativa*) that was formulated to meet the goats’ feeding requirements according to NRC [29]. Goats were housed in shaded pens with free access to water. The first group (C) received the basal diet with no additives, the second group (T) received the basal diet supplemented with commercial Quebracho tannins extract (10 g/head/day), and the third group (HM) received the basal diet supplemented with the herbal mixture (10 g/head/day). The herbal mixture contained 25% ginger (*Zingiber officinale*), 25% garlic (*Allium sativum*), 25% artemisia (*Artemisia vulgaris*), and 25% turmeric (*Curcuma longa*) that were thoroughly mixed in equal quantities. Animals received the condensed tannin and herbal mixture gradually at 1% of their dry matter (DM) feed intake. Table 1 shows details of the components and chemical compositions of the experimental diet. The weights of the goats were recorded at the beginning and the end of the trial. Drinking water was offered twice a day, the goats were housed in goat pens, and the trial period was 90 days. All experimental additives were mixed with concentrate to confirm full intake. Orts were weighed and feed intake was recorded daily. Diet and orts were sampled weekly and dried in a forced-air oven at 65 ^o^ C for 48 h. At the end of the study four animals from every group were slaughtered to conduct the histological evaluation and the rest of animals were released to the goats herd.

**Table 1:** Chemical analysis of experimental diets (%)

Item	DM	OM	Ash	EE	CP	NDF	ADF
Alfalfa (*Medicago sativa*) Hay	89	84.24	15.76	1.81	15.5	58.45	42.15
Concentrate feed mixture*	90	91.14	8.86	2.92	16.5	29.15	7.84

*Concentrate feed mixture consisted of corn 60%, soybean meal 12%, wheat bran 12%, barley 8%,

cotton meal 5%, lime stone 1.3%, salt 0.5%, Sodium bicarbonate 0.3%, Trace Minerals 0.4%,

Vitamins 0.3%, Antitoxins 0.2%. DM = Dry matter; OM = Organic matter; CP = Crude protein; EE = Ether extract; NDF = Neutral detergent fiber; ADF = Acid detergent fiber

### Rumen fermentation and predicted methane production

Rumen samples were collected from the animals using a stomach tube and the pH was measured immediately using a pH meter. The rumen contents were filtered through 4 layers of cheesecloth, and rumen fluid samples were used to measure VFA and ammonia as well as DNA extraction to study the microbial community. To analyze ammonia and VFA, 1 mL of rumen fluid samples were transferred to 1.5 mL Eppendorf tubes and acidified with 200 µL of meta-phosphoric acid 25% (w/v). The samples were then stored at -20 °C for later analysis. After thawing, the samples were centrifuged at 30,000×g (15,000 rpm, JA-17 rotor) for 20 min and the resulting supernatant was used for VFA and ammonia determination. For rumen ammonia nitrogen (NH_3_-N) measurement, 250 µL of supernatant was assessed calorimetrically using an ammonia assay kit (Biodiagnostic, Cairo, Egypt). The remaining supernatants (750 µL) were transferred to GC vials for VFA analysis using a capillary column (TR-FFAP 30 m×0.53 mmI D×0.5 μm) in a Thermo Scientific TRACE 1300 gas chromatography system (Thermo Scientific, Massachusetts, United States). The oven temperature was ramped from 100 to 200 °C at a rate of 10 °C/min, while the injection and flame ionization detector (FID) temperatures were set at 220 and 250 °C, respectively. Nitrogen was used as the carrier gas at a flow rate of 7 ml/min, while hydrogen and make-up gases were set at flow rates of 40 ml/min and 35 ml/min, respectively. The total run time was set at 10 min with calibration done using a standard with known concentrations of VFA. The predicted methane was calculated using the concentration of propionic acid, Methane yield = 316/propionate + 4.4, according to Williams et al. [30].

### Histomorphological examination of rumen samples

At the end of the experiment, four goats from each group were selected randomly to slaughter. Animals were fasted for 12 h with ad libitum access to water, transported to Maryout Research Station’s slaughterhouse, and weighed before the slaughter. The slaughter was conducted by an experienced technician by severing the jugular vein with a sharp knife without electrical stimulation. The death of the animals was ensured prior to further processing and sampling. After bleeding, skinning, and eviscerations, rumen autopsy samples from slaughtered goats were collected to conduct the histological examination. The samples were collected and fixed with a 10% neutral buffered formalin solution, then washed, dehydrated in ethyl alcohol of different grades, cleared in methyl benzoate, and embedded in paraffin wax. Blocks were processed using standard procedures, and 5-µm sections were stained with hematoxylin and eosin and examined microscopically [31].

### Chemical composition

Dried feeds and orts were ground and analyzed according to the method of AOAC [32] to measure DM, crude Protein (CP), and ether extract (EE). Neutral detergent fiber (NDF) and acid detergent fiber (ADF) were measured using ANKOM 200 Fiber Analyzer (ANKOM Technology, New York, United States) according the method of Van Soest et al. [33]. Total phenolic, total flavonoids, total tannins, and total saponins were determined in the herbal mixture. Total flavonoids were extracted using petroleum ether and 95% ethanol and determined according to the method of Karawaya and Aboutabl [34]. Total phenolic content was determined by the Folin–Ciocalteu according to the method of Kaur and Kapoor [35]. Total saponins were determined using 70% ethanol extraction according to the method of Kurkin and Ryazanova [36]. Total tannins were extracted by boiling in water according to the method of Balbaa [37]. Flavonoids compounds were determined using high-performance liquid chromatography (HPLC) (Thermo Scientific, Massachusetts, United States) using reversed-phase C18 column and 0.05%Trifluoroacetic acid/Acetonitrile (solvent A) and distilled water (solvent B) as a mobile phase [38].

### Microbial community

#### DNA isolation and PCR amplification

Total DNA was extracted from 500 µl of rumen samples. Subsequently, the sample was centrifuged at 13,000 rpm, and the pellets were used in DNA extraction using the QIAamp DNA Stool Mini Kit (Qiagen, Hilden, Germany) according to the manufacturer’s instructions. The quality and quantity of DNA were assessed using gel electrophoresis and a Nanodrop spectrophotometer 2000 (Thermo Scientific, Massachusetts, United States). The composition and diversity of rumen bacterial and archaeal communities were investigated using amplification of the variable V4 region on 16 S rDNA. The rumen bacterial community was studied by amplification of V4 region by 515 F and 926R primers using the following PCR conditions: 94 °C for 3 min; 35 cycles of 94 °C for 45 s, 50 °C for 60 s, and 72 °C for 90 s; and 72 °C for 10 min. Rumen archaeal community was studied by the amplification of the V4 region using primers Ar915aF (5-AGGAATTGGCGGGGGAGCAC-3) and Ar1386R (5-GCGGTGTGTGCAAGGAGC-3) [6]. The PCR amplification was conducted under the following conditions: 95 °C for 5 min; 30 cycles of 95 °C for 20 s, 55 °C for 15 s, 72 °C for 5 min, and 72 °C for 10 min. PCR amplicons were purified and sequenced using the Illumina MiSeq system (Illumina, California, United States).

### Bioinformatics and statistical analyses

The generated paired-end sequence reads were analyzed using the DADA2 pipeline through the R platform [39]. The fastq files of sequence reads were demultiplexed, and their quality was evaluated. Then, the sequences were filtered, trimmed, and dereplicated followed by merging read 1 and read 2 together to get denoised sequences. The chimeras were removed from the denoised sequences to generate Amplicon Sequence Variants (ASVs). Taxonomic assignment of ASVs was performed using the “*assignTaxonomy*” and “*addSpecies”* functions, and microbial taxa were identified using the SILVA reference database (version 138). Alpha diversity metrics, including observed ASVs, Chao1, Shannon, and Inverse Simpson, were measured. In addition, the Beta diversity of bacterial and archaeal communities was determined as principal coordinate analysis (PCoA) using Bray–Curtis dissimilarity and figures were created using the phyloseq and ggplot packages. The raw sequence reads were deposited to SRA at https://www.ncbi.nlm.nih.gov/sra/PRJNA1008569.

### Statistical analysis

All the data were analyzed using one-way ANOVA in IBM SPSS software v. 20.0 [40] using the Duncan test. And the differences at *P* < 0.05 were considered statistically significant. Pearson correlation analysis (Heatmap) and Principal component analysis (PCA) were conducted between Relative Growth Rate (RGR), feed intake, predicted methane, rumen fermentation parameters, and relative abundances of dominant bacterial phyla, and dominant bacterial and archaeal genera.

## Results

### Phytochemical substances in the herbal mixture (HM)

The phytochemical compounds in the HM mixture consisted of total phenolics 60 mg/100 g, total flavonoids 17.63 mg/100 g, total Saponins 48.8 mg/100 g, and total tannins 49 mg/100 g. Flavonoid compounds consisted of Apeginin (20.07% of flavonoids), Rutin (0.18%), Diosmin (5.25%), Kampferol (74.5%).

### Feed intake and growth performance

Table 2 presents the relative growth rate (RGR) and feed intake expressed as dry matter intake (DMI), organic matter intake (OMI), crude protein intake (CPI), ether extract intake (EEI), and neutral detergent fiber intake (NDFI) calculated as g/kg ^0.75^ (kilogram metabolic body weight) as affected by feeding treatments. Feed intake was similar between groups and group HM showed higher numeric RGR without significant differences (Table 2). On the other hand, tannin and herbal mixture intake represented 1% of DM intake.

**Table 2:** Effects of dietary tannin and herbal mixture supplementation on growth performance and feed intake in goats

	Treatments			Mean	SEM	P-value
	C	T	HM			
Initial body Weight, Kg	27.67	26.1	26.1	26.55	1.33	0.88
%RGR*	27.98	32.15	34.94	31.95	3.40	0.75
Feed Intake, g/Kg ^0.75^						
Dry matter	85.36	80.57	81.57	82.30	1.32	0.35
Organic matter	75.61	71.36	72.25	72.89	1.17	0.35
Crude protein	15.76	14.88	15.06	15.20	0.24	0.35
Ether Extract	2.13	2.01	2.04	2.06	0.033	0.35
Neutral detergent fiber intake	34.19	32.27	32.68	32.97	0.53	0.35

*Relative Growth Rate (RGR), % = (final BW – initial BW) × 100/IBW

C = diet without supplementation; T = diet supplemented with tannins;

HM = diet supplemented with herbal mixture; SEM = standard error of the mean

### Rumen fermentation

Rumen pH and ammonia were similar between goat groups (*P* > 0.05) (Table 3) and the pH was < 6 in group C. The production of total VFA (TVFA) was higher in herbal mixture-supplemented group (HM) and the C group showed the lowest production (*P* < 0.05) (Table 3). Similar trends were obtained in the proportions of acetic, butyric, and isobutyric (*P* < 0.05). The values of propionic, valeric, and isovaleric were similar in the experimental groups (Table 3). Based on the results of VFA profile, predicted methane was declined (*P* < 0.05) due to the supplementation of tannin and herbal mixture.

**Table 3:** Effects of tannin and herbal mixture supplementation on rumen fermentation parameters and predicted methane in goats

	Treatments			Mean	SEM	P-value
	C	T	HM			
pH:	5.85	6.5	6.16	6.19	0.221	0.53
Total VFA, mM:	91.16^a^	104.55^ab^	116.81^b^	105.10	3.77	0.01
Ammonia mg/dl	19.63	21.71	19.55	20.35	1.40	0.79
Acetic, mM	55.09^a^	65.05^ab^	72.89^b^	65.00	2.72	0.018
Propionic, mM	18.98	21.19	23.64	21.43	0.91	0.12
Isobutyric, mM	0.54^a^	0.70^b^	0.71^b^	0.65	0.030	0.046
Butyric, mM	14.59^a^	15.35^ab^	17.27^b^	15.82	0.47	0.042
Isovaleric, mM	0.72	1.00	1.05	0.94	0.12	0.57
Valeric, mM	1.23	1.23	1.25	1.24	0.05	0.99
Predicted methane, g /kg DMI	23.15^b^	19.40^a^	17.21^a^	19.70	0.77	0.001

VFA = volatile fatty acids. C = diet without supplementation; T = diet supplemented with tannins;

HM = diet supplemented with herbal mixture; SEM = standard error of the mean

### Analysis of microbial communities

#### Diversity of bacterial community

**Fig. 1:**
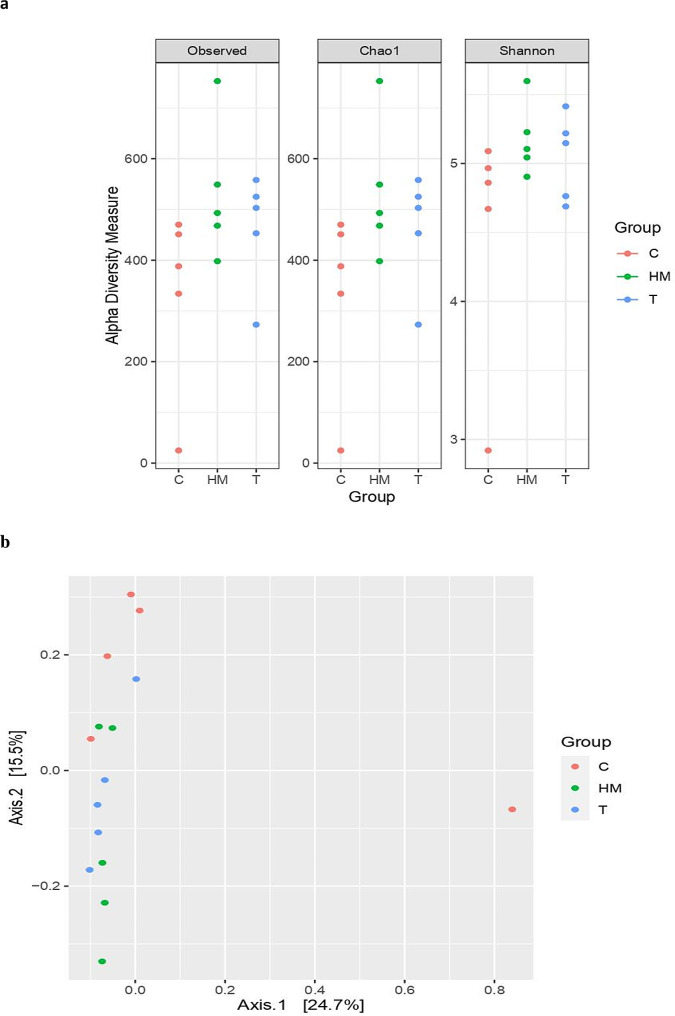
Alpha diversity indices (**a**) and Principal coordinates analysis (PCoA) (**b**) of bacterial community. Alpha diversity indices, including observed species, Chao1, and Shannon as well as PCoA analysis based on Bray-Curtis dissimilarity. The analyses were performed between three goat groups: red circles for the control group (**C**), blue circles for the tannin-supplemented group (T), and green circles for the herbal mixture-supplemented group (HM)

The amplicon sequencing of 16 S rDNA genes revealed a total of 421,540 high-quality non-chimeric sequences read with an average of 25,949 ± 2241 sequences per sample. No significant differences in the number of Amplicon Sequence Variants (ASVs) and alpha diversity indexes, Chao, Shannon, and invsimpson across dietary treatments (Table 4; Fig. 1a). Beta diversity was estimated and visualized for the bacteria community of goats under investigation using principal coordinate analysis (PCoA) based on Bray-Curtis dissimilarity (Fig. 1b). The plot showed that the samples of control groups (C) were separated clearly from the supplemented groups (T and HM).

**Table 4:** Effects of dietary tannin and herbal mixture supplementation on alpha diversity indices of rumen bacterial community in goats

	Treatments			Mean	SEM	P-value
	C	T	HM			
Average Sequences reads	24,477	28,158	24,919	25,949	2241	0.79
Observed ASVs	410.75	462.4	532.2	468.45	28.62	0.23
Chao1	410.75	462.4	532.2	468.45	28.62	0.23
Shannon	4.89	5.04	5.17	5.03	0.06	0.25
Invsimpsone	53.58	72.20	79.92	68.56	7.17	0.32

C = diet without supplementation; T = diet supplemented with tannins;

HM = diet supplemented with herbal mixture; SEM = standard error of the mean

### Composition of the bacterial community

The taxonomic analysis of the bacterial communities in the rumen of goats under study revealed a total of 15 bacterial phyla, out of which one phylum (Synergistota, 0.28%) was detected only in the HM group (Table 5). Bacterial phyla that represented more than 1% of the bacterial community were Bacteroidota (54.62%), Cyanobacteria (1.33%), Firmicutes (40.29%), and Planctomycetota (1.50%). Furthermore, bacterial phyla that represented less than 1% of the bacterial community were Actinobacteriota (0.31%), Chloroflexi (0.15%), Desulfobacterota (0.08%), Elusimicrobiota (0.058%), Fibrobacterota (0.07%), Proteobacteria (0.23), Spirochaetota (0.69%), and Verrucomicrobiota (0.66%) (Table 5). The dietary supplementation affected the relative abundance of six bacteria phyla, including, Actinobacteriota, Bacteroidota, Elusimicrobiota, Firmicutes, Proteobacteria, and Verrucomicrobiota (Table 5).

**Table 5:** Effects of dietary tannin and herbal mixture supplementation on relative abundances (%) of rumen bacterial phyla in goats

	Treatments			Mean	SEM	P-value
	C	T	HM			
Actinobacteriota	0.79^b^	0.05^a^	0.10^a^	0.31	0.14	0.048
Bacteroidota	38.88^a^	62.37^b^	62.62^b^	54.62	3.62	0.001
Chloroflexi	0.23	0.09	0.149	0.15	0.028	0.12
Cyanobacteria	1.42	0.86	1.72	1.33	0.21	0.27
Desulfobacterota	0.11	0.05	0.09	0.08	0.02	0.54
Elusimicrobiota	0.09^b^	0.04^a^	0.036a	0.058	0.01	0.03
Fibrobacterota	0.09	0.064	0.05	0.07	0.014	0.39
Firmicutes	55.56^b^	34.54^a^	30.77^a^	40.29	3.37	0.0001
Planctomycetota	1.86	0.79	1.86	1.50	0.33	0.34
Proteobacteria	0.14^a^	0.21^a^	0.34^b^	0.23	0.030	0.012
Spirochaetota	0.70	0.46	0.92	0.69	0.12	0.32
Verrucomicrobiota	0.47^a^	0.42^a^	1.09^b^	0.66	0.12	0.026
Synergistota	0	0	0.28	0	0	0

C = diet without supplementation; T = diet supplemented with tannins;

HM = diet supplemented with herbal mixture; SEM = standard error of the mean

Phylum Bacteroidota dominated the bacterial community and its relative abundance was increased due to dietary supplementation, whenever the goat group HM showed the highest relative abundance (62.62%) and group C showed the lowest relative abundance (Table 5). This phylum was dominated by family Prevotellaceae, F082, Bacteroidales RF16 group, Muribaculaceae, Bacteroidales BS11 gut group, Rikenellaceae, and Bacteroidales UCG-001. The relative abundance of those families was improved by dietary supplementation (Table 6).

**Table 6:** Effects of dietary tannin and herbal mixture supplementation on relative abundances (%) of dominant rumen bacterial genera in goats

	Treatments					Mean		SEM	P-value
	C		T	HM					
P: Bacteroidota									
F: Prevotellaceae	24.55^a^		41.66^b^	40.37^b^	35.53		2.98		0.019
F: Prevotellaceae; G: Prevotella	11.94^a^		22.04^b^	19.36^b^	17.78		1.70		0.028
F: Bacteroidales RF16 group; G: NA	2.26^a^		5.33^ab^	10.42^b^	6.00		1.34		0.029
F: Prevotellaceae; G: Prevotellaceae UCG-001	2.05		1.69	2.24	1.99		0.22		0.64
F: F082; G: NA	9.42		17.96	16.02	14.47		1.59		0.06
F: Prevotellaceae; G: Prevotellaceae UCG-003	0.52^a^		0.99^a^	3.54^b^	1.68		0.45		0.004
F: Prevotellaceae; G: Alloprevotella	0		0.05	0.08	0		0		0
F: Muribaculaceae: G: NA	3.53		4.98	4.49	4.34		0.57		0.60
F: Bacteroidales BS11 gut group; G: NA	0.31		0.32	0.50	0.37		0.07		0.56
F: Rikenellaceae; G: Rikenellaceae RC9 gut group	5.66^a^		9.13^b^	9.33^b^	8.04		0.63		0.016
F: Rikenellaceae	5.83^a^		9.49^b^	10.04^b^	8.46		0.65		0.005
F: Bacteroidales UCG-001; G: NA	0.03		0.03	0.04	0.04		0.005		0.83
P: Chloroflexi									
F: Anaerolineaceae; G: Flexilinea	0.23		0.09	0.14	0.15		0.03		0.12
P: Firmicutes									
F: Ruminococcaceae		25.43^b^	11.98^a^	6.89^a^	14.77		2.39		0.0001
F: Lachnospiraceae		2.91^b^	1.72^a^	1.91^a^	2.18		0.20		0.024
F: Lachnospiraceae; G: Lachnospiraceae ND3007 group		0.58^b^	0.15^a^	0.17^a^	0.30		0.06		0.004
F: Lachnospiraceae; G: Acetitomaculum		1.24	0.33	0.21	0.59		0.20		0.06
F: Lachnospiraceae; G: Butyrivibrio		0.20	0.10	0.20	0.17		0.02		0.23
F: Lachnospiraceae; G: Marvinbryantia		0	0.04	0.06	0		0		0
F: Christensenellaceae		9.69	7.29	6.59	7.85		0.76		0.23
F: Christensenellaceae; G: Christensenellaceae R-7		9.54	7.18	7.08	7.93		0.68		0.27
F: Oscillospiraceae		7.53	8.66	9.38	8.52		0.63		0.52
F: Oscillospiraceae; G: UCG-002		0.16^a^	0.63^ab^	0.95^b^	0.58		0.12		0.027
F: Oscillospiraceae; G: NK4A214 group		6.97	7.31	7.81	7.36		0.58		0.85
F: Oscillospiraceae; G: UCG-005		0.08^a^	0.22^ab^	0.27^b^	0.19		0.03		0.049
F: Oscillospiraceae; G: Papillibacter		0	0.08	0.06	0		0		0
F: Hungateiclostridiaceae; G: Saccharofermentans		0.43^b^	0.12^a^	0.20^a^	0.25		0.04		0.002
F: Selenomonadaceae		0.14	0.66	0.62	0.47		0.10		0.05
F: Selenomonadaceae; G: Veillonellaceae UCG-001		0.01	0.04	0.06	0.04		0.018		0.18
F: Selenomonadaceae; G: Anaerovibrio		0	0.05	0.05	0		0		0
F: Anaerovoracaceae; G: Mogibacterium		0.42^b^	0.07^a^	0.12^a^	0.201		0.05		0.002
F: Monoglobaceae; G: Monoglobus		0.14	0.09	0.10	0.11		*0.02*		0.50
F: Acidamino:coccaceae; G: Succiniclasticum		0.02	0.06	0.13	0.07		0.02		0.31
F: Streptococcaceae; G: Streptococcus		0.08	0.06	0.06	0.06		0.01		0.75
F: Acholeplasmataceae; G: Anaeroplasma		0.02^a^	0.03^a^	0.08^b^	0.047		0.01		0.03
P: Planctomycetota									
F: Pirellulaceae; G: p-1088-a5 gut group		0.68^a^	0.72^a^	1.67^b^	1.02		0.19		0.049
P: Spirochaetota									
F: Spirochaetaceae; G: Sphaerochaeta		0.18^a^	0.30^a^	0.61^b^	0.36		0.06		0.004
F: Spirochaetaceae; G: Treponema		0.32	0.20	0.31	0.27		0.03		0.34

C = diet without supplementation; T = diet supplemented with tannins;

HM = diet supplemented with herbal mixture; SEM = standard error of the mean

On the genus level, the phylum Bacteroidota is dominated by genus *Prevotella*, *Prevotellaceae UCG-001*, and Rikenellaceae *RC9* gut group. In addition, genus *Alloprevotella* was detected only in T and HM groups. Members of phylum Firmicutes were classified into family Ruminococcaceae, Lachnospiraceae, Christensenellaceae, Hungateiclostridiaceae, Anaerovoracaceae, Monoglobaceae, and Streptococcaceae that were declined in groups T and HM (Table 6). In addition to families Oscillospiraceae, Selenomonadaceae, Monoglobaceae, Acidamino:coccaceae, and Acholeplasmataceae whose relative abundances were increased in T and HM supplementation (Table 6).

On the genus level, phylum Firmicutes was affiliated with *Lachnospiraceae ND3007* group, *Acetitomaculum*, *Butyrivibrio*, *Christensenellaceae R-7* group, *Saccharofermentans*, *Mogibacterium*, *Monoglobus*, and *Streptococcus* that were declined in T and HM. In addition to genus *UCG-002*, *NK4A214* group, *UCG-005*, *Veillonellaceae UCG-001*, *Succiniclasticum*, and *Anaeroplasma* that were increased in T and HM (Table 6). Furthermore, some genera within Firmicutes were detected only in T and HM such as *Marvinbryantia*, *Papillibacter*, and *Anaerovibrio*. Phylum Planctomycetota was dominated by family Pirellulaceae and genus *p-1088-a5* gut group that showed its highest relative abundance in the HM group (Table 6). Phylum Spirochaetota was dominated by family Spirochaetaceae that classified into genus *Sphaerochaeta* and *Treponema* which were increased by the supplementation (Table 6).

### Diversity of the archaeal community

**Fig. 2:**
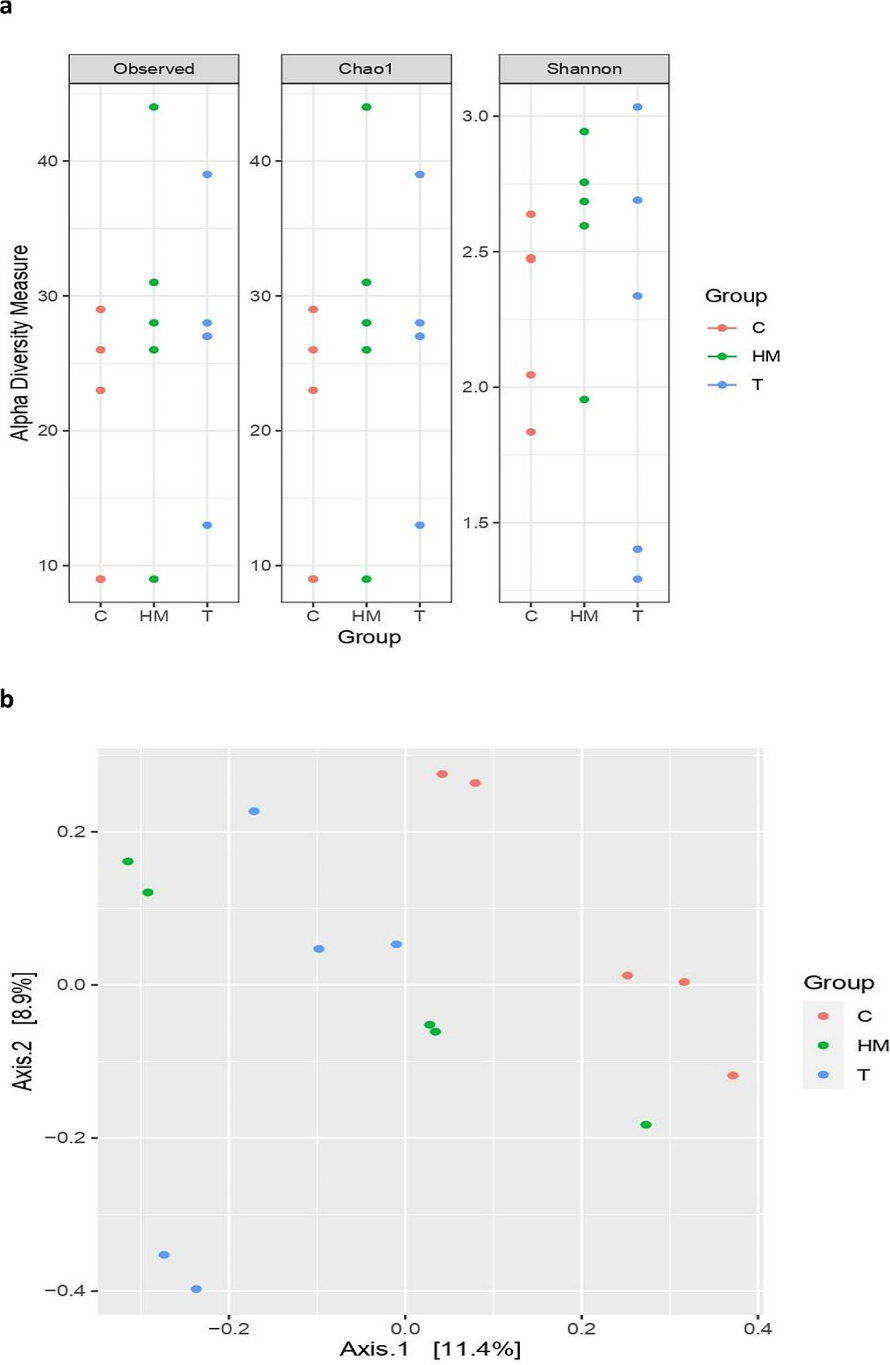
Alpha diversity indices (**a**) and Principal coordinates analysis (PCoA) (**b**) of archaeal community. Alpha diversity indices, including observed species, Chao1, and Shannon as well as PCoA analysis based on Bray-Curtis dissimilarity. The analyses were performed between three goat groups: red circles for the control group (**C**), blue circles for the tannin-supplemented group (T), and green circles for the herbal mixture-supplemented group (HM)

The amplicon sequencing of archaeal 16 S rDNA genes revealed 178,388 high-quality reads with an average of 11,880 reads per sample. Furthermore, 368 ASVs were detected with an average of 25 ASVs per sample (Table 7) (Fig. 2a). The feeding treatment did not affect the alpha diversity metrics, observed ASVs, Chao, Shannon, and invsimpson (Table 7). Principal coordinate analysis (PCoA) based on Bray-Curtis dissimilarity (Fig. 2b) was used to estimate and visualize the beta diversity of archaeal communities in the rumen of goats. The plot revealed that samples of the C group were separated from the samples of supplemented groups (T and HM).

**Table 7:** Effects of dietary tannin and herbal mixture supplementation on alpha diversity indices and relative abundances (%) of rumen archaea in goats

	Treatments			Mean	SEM	P-value
	C	T	HM			
Alpha diversity						
Sequences reads:	8606	8127	18,253	11,880	2411	0.14
Observed ASVs	19.2	27	27.6	25	2.71	0.40
Chao1	19.2	26.8	27.6	24.53	2.71	0.40
Shannon	2.29	2.15	2.58	2.34	0.13	0.44
Invsimpsone	7.19	6.54	9.47	7.73	0.96	0.46
The relative abundances of archaeal genera (%)						
P:Euryarchaeota; F: Methanobacteriaceae						
Methanobrevibacter	99.00^b^	98.80^b^	96.69^a^	98.16	0.36	0.006
Methanosphaera	0	0	0.87	0	0	0
P: Thermoplasmatota; F: Methanomethylophilaceae						
Unclass_Methanomethylophilaceae	0.81	0.89	1.88	1.20	0.21	0.05
G: Candidatus Methanomethylophilus	0.17^a^	0.16^a^	0.45^b^	0.26	0.04	0.01
P: Halobacterota						
F: Halococcaceae; G: Halococcus	0	0	0.08	0	0	0
F: Haloferacaceae; G: Natronococcus	0	0	0.06	0	0	0

C = diet without supplementation; T = diet supplemented with tannins;

HM = diet supplemented with herbal mixture; SEM = standard error of the mean

### Composition of archaeal community

The taxonomic analysis of the archaeal community in goat groups showed that the community was affiliated to three phyla, Euryarchaeota, Thermoplasmatota, and Halobacterota (Table 7). Phylum Euryarchaeota was dominated by Methanobacteriaceae, which was classified into genus *Methanobrevibacter* that was declined by supplementation (*P* < 0.05), and genus *Methanosphaera*, which was only detected in the HM group (Table 7). Phylum Thermoplasmatota was dominated by family Methanomethylophilaceae that had two genera, *Unclass_Methanomethylophilaceae* and *Candidatus Methanomethylophilus* that showed their highest relative abundances in group HM group (*P* < 0.05). Phylum Halobacterota was classified into families Halococcaceae and Haloferacaceae which were further classified into genus *Halococcus* and *Natronococcus*, respectively. Both *Halococcus* and *Natronococcus* were detected only in the HM group (Table 7).

### Pearson correlation analysis

**Fig. 3:**
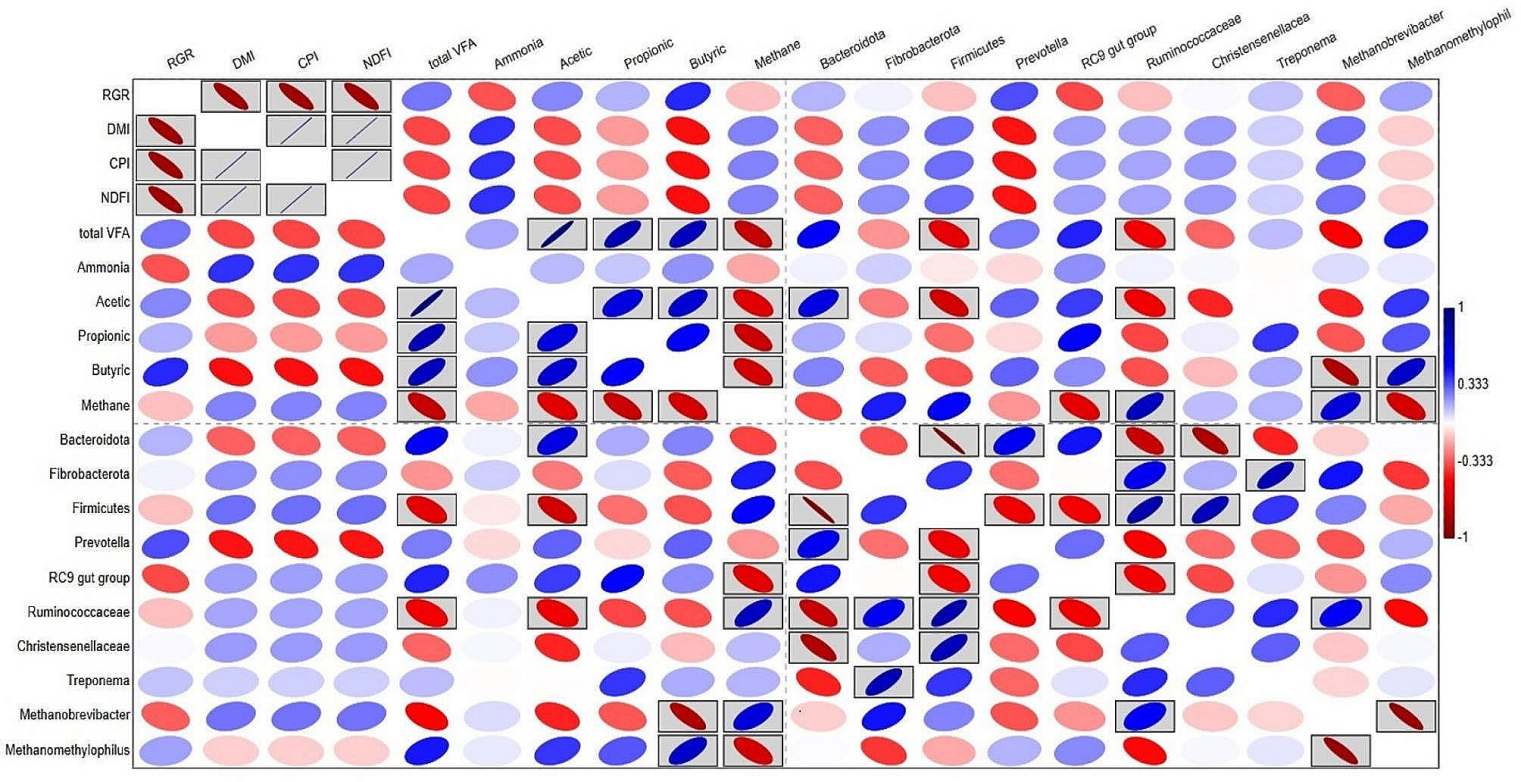
Heatmap based on Pearson correlation. The correlation was conducted between the RGR, feed intake, rumen fermentation parameters, predicted methane production, and relative abundances of dominant ruminal bacteria and archaea of goats fed different diets. The black boxed ellipses indicate to significant correlations at *P* < 0.05

Pearson correlation analysis (Fig. 3) revealed several positive and negative relationships. Relative growth rate (RGR) correlated negatively with feed intake (DMI, CPI, and NDFI), rumen ammonia, and methane, and the relative abundances of *Rikenellaceae RC9 gut group*, and *Methanobrevibacter*. In addition, RGR correlated positively with total VFA, butyric, and the relative abundances of *Prevotella*, and *Methanomethylophilus*. A negative correlation was observed between *Methanobrevibacter* and total VFA. *Methanobrevibacter* correlated positively with methane production.

### Effect of the dietary supplementation on performance, rumen fermentation and relative abundance of rumen bacteria and archaea

**Fig. 4:**
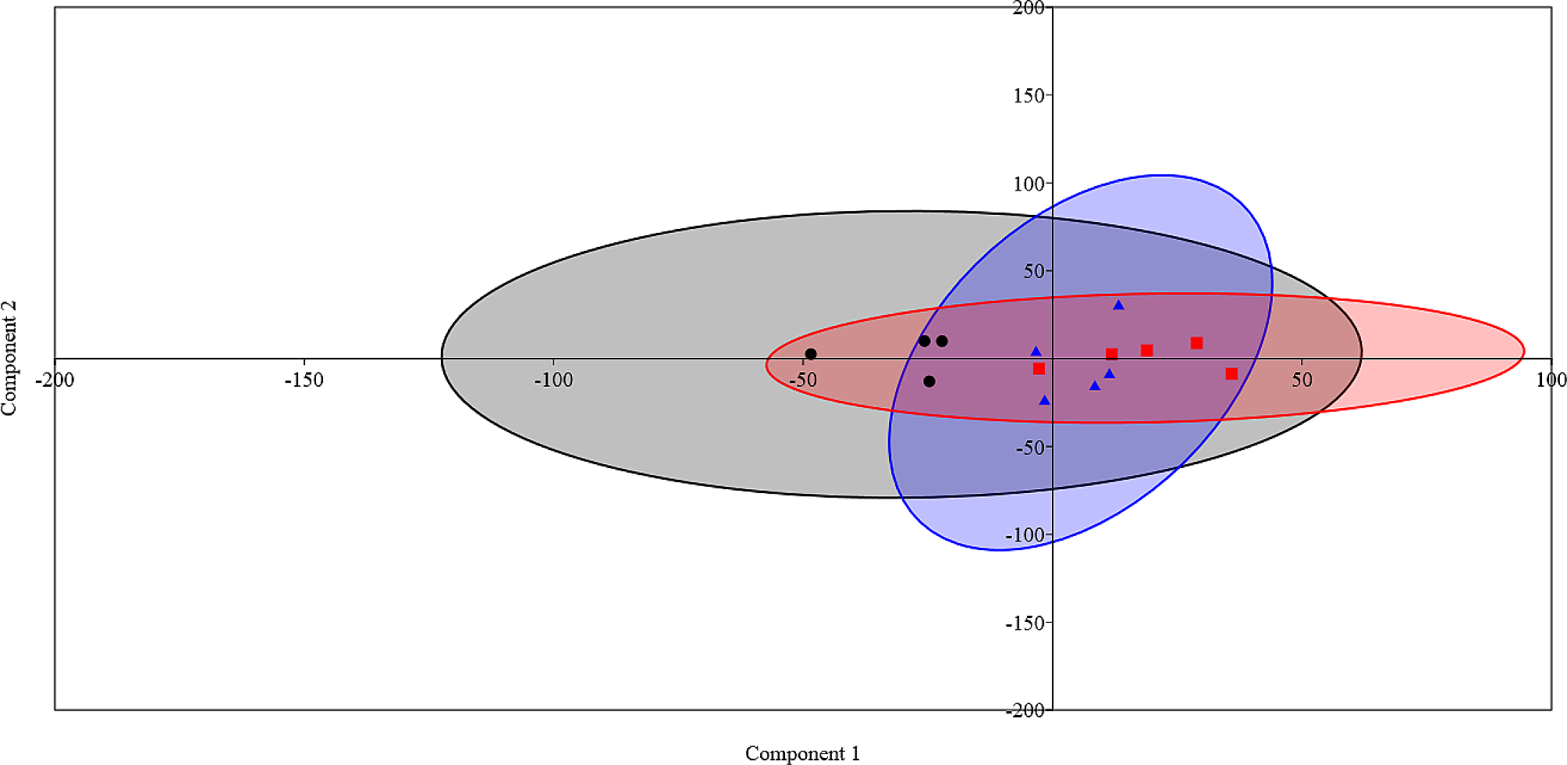
Principal component analysis (PCA). The PCA was conducted using the data of the RGR,feed intake,rumen fermentation parameters, predicted methane production, and relative abundances of dominant ruminal bacteria and archaea of goats fed different diets. Black dots are for group C, blue triangles are for group T, and red squares are for group HM

Principal component analysis (PCA) (Fig. 4) was conducted based on RGR, feed intake, methane, rumen fermentation parameters, and the relative abundance of dominant bacteria and archaea. The PCA plot showed that the samples were separated into three groups. The important parameters that drove the differences between the samples were RGR, total VFA, acetic, the relative abundances of Firmicutes, *Prevotella*, and *Ruminococcaceae*.

#### Histological examination of the rumen

**Fig. 5:**
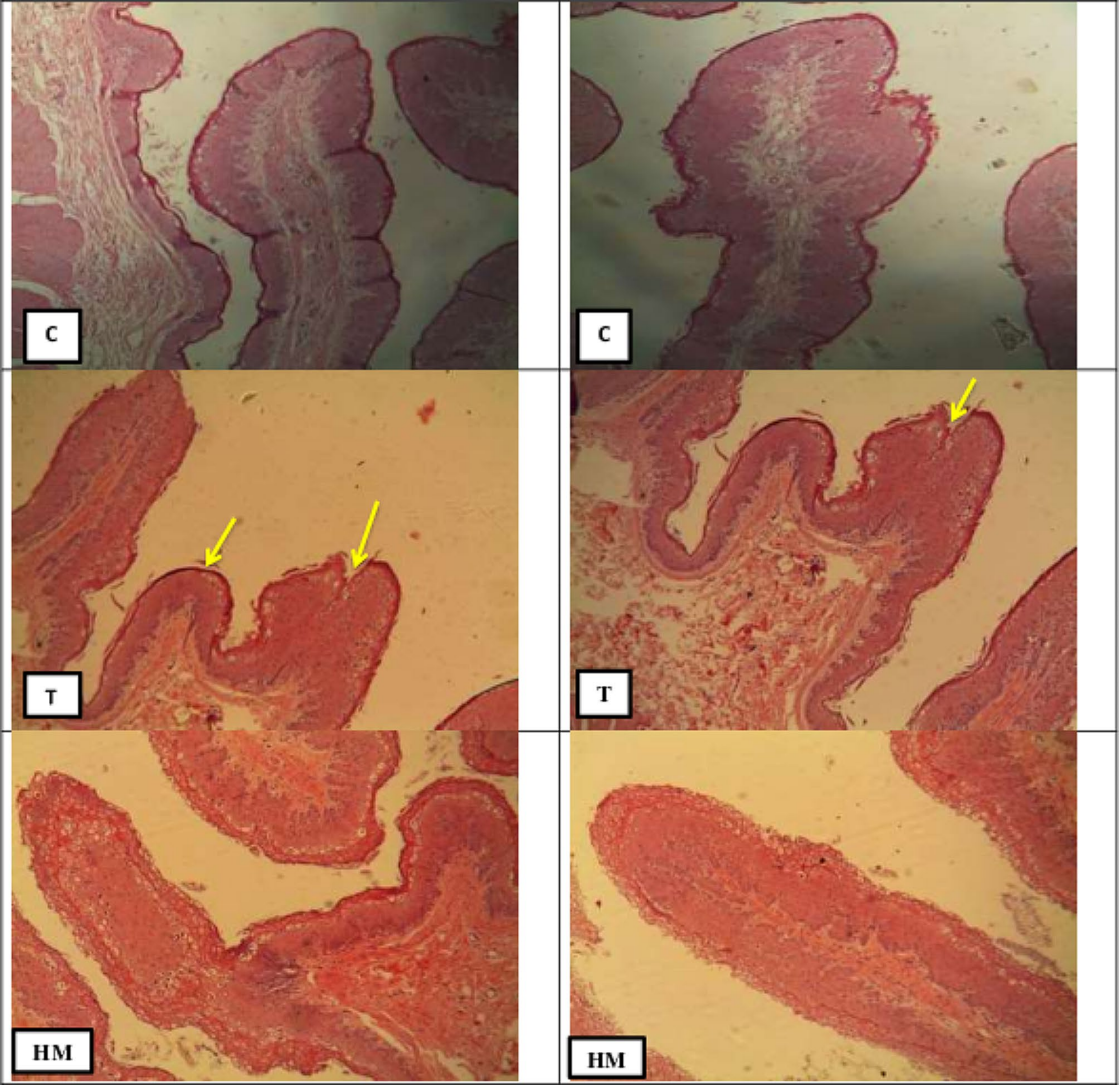
The histology of goat rumen papillae as affected by tannin and herbal supplementation. C: refers to rumen papillae of the control group and showing normal ruminal papillae, intact mucosa, sub mucosa and musclosa (H&E, X10); T refers to ruminal papillae of the tannin-supplemented group and showing degeneration and atrophy of ruminal papillae, shortness of papillae and rupture (yellow arrows); HM refers to papillae of the herbal mixture-supplemented group and showing normal histological appearance

Examination of the rumen sector from group C and HM showed a normal appearance of ruminal papillae and musclosa; while, shortness of ruminal papillae, slight degeneration, and atrophy were detected in group TT (Fig. 5).

## Discussion

### Animal performance

Phytogenic substances such as phenolics, flavonoids, Saponins, and tannins are promising modifiers for the rumen microbial community to improve rumen fermentation and animal performance [21, 23], which highlights the herbal mixture used in the current study as it contains a variety of phytochemicals that provide the synergistic effect to the animal [41–43]. Previous studies [28, 44, 45] on herbal mixtures reported no changes in animal feed intake, which support the current findings. In addition, Reynolds et al. [46] included tannin-rich ground pine bark in the diets of meat goats and observed no changes in feed intake and daily gain. Glasscock et al. [47] reported that herbal plants mixture did not affect DMI or RGR in goats. In contrast, Waghorn et al. [48] reported that high tannin concentration in animal diets depressed the digestibility and declined animal feed intake. In the current study, the concentration of condensed tannin was 1% of DM feed intake and did not affect feed intake or RGR negatively, which indicates that tannins concentration is in the suitable range. This speculation is supported by Min et al. [49] who suggested that 2–4% tannin based on DM is suitable to maintain favorable growth performance and rumen fermentation. Orzuna-Orzuna et al. [45] explained that flavonoids supplementation modulates and improves rumen fermentation and metabolism to improve animal performance and health in ruminants.

### Rumen fermentation parameters

Rumen pH was > 6 in group T and HM, which supports the fiber degradation, and VFA production in the rumen [50], which could explain higher VFA production in T and HM [44, 45]. Li et al. [23] supplemented lactating buffalo with flavonoids and noticed improvements in the VFAs’ production, which support the current findings. The higher acetic acid in supplemented groups was also indicated in cattle supplemented with ensiled mulberry leaves [51]. Razo Ortiz et al. [28] explained that polyherbal mixtures stimulated the propionic and butyric acids-producing bacteria such as *Prevotella* and *Butyrivibrio* which improve the VFA production, and this explanation is supported by the correlation between rumen bacteria and rumen fermentation parameters (Fig. 3).

### Bacterial community

The improvements in the rumen fermentation parameters were consistent with variations in the composition of microbial communities due to the supplementation, which agrees with previous studies [44, 52]. A large proportion of the bacterial community was affiliated with the members of phylum Bacteroidota and Firmicutes, which agrees with previous studies on rumen bacteria of goats [45, 53, 54]. The supplementation increased the members of Bacteroidota and Proteobacteria and declined the members of Firmicutes and Actinobacteriota (Table 5). Li et al. [23] noticed that flavonoids supplementation in lactating buffalo increased Bacteroidota, Firmicutes, and Proteobacteria, but declined Actinobacteriota. On the other hand, Rabee et al. [26] reported that phylum Fibrobacteria is sensitive to phenolic compounds, which explains its decline in the supplemented groups (T, HM). The members of Bacteriodota degrade wide range of substrates including cellulose, pectin, and soluble polysaccharides and unclassified members of this phylum are specialized in lignocellulose degradation [26]. The main members of this phylum, *Prevotella* and *Rikenellaceae RC9* gut group, were higher in supplemented groups (T and HM) (Table 6), which is supported by previous studies [23, 52, 55].

Genus *Prevotella* dominated the bacterial communities in several ruminant species and it digests wide range of substrates such as hemicellulose and protein [56]. Furthermore, this genus is the main propionate producers in the rumen [57], which explains the positive correlation between *Prevotella* and propionic (Fig. 3). Furthermore, *Prevotella* was associated with high efficient cattle [58], which support the correlation between RGR and genus *Prevotella*. Li et al. [23] reported higher *Prevotella* in Buffalo supplemented with Flavonides, which supports the current finding. Genus *Rikenellaceae RC9* gut group, the second dominant genus in phylum Bacteriodota, has an important role in fiber degradation in the rumen [59, 60]. The increment in the *Prevotella* and *Rikenellaceae RC9* gut group is a positive point for the supplementation, which is supported by the positive correlation between RGR and *Prevotella*, Bacteriodota, total VFA, and propionic (Fig. 3).

On the other hand, the prevalence of *Prevotella* and *RC9_*gut_group in the rumen of goats supplemented with tannin and herbal mixture highlights the resistance of these genera to phytochemicals [26]. Genus *Alloprevotella* is well adapted to tannin and other plant secondary metabolites, which explains its presence in group T and HM [26]. Hassan et al. [25] explained that essential oils of *Origanum vulgare*, garlic and peppermint decreased the abundance of Firmicutes and methane production, while increasing Bacteroidetes, which support the current findings. Family Ruminococcaceae, the dominant family in Firmicutes, includes cellulolytic bacteria and is sensitive to different types of tannins, which could justify the decline in the relative abundance of this family in the rumen of the supplemented goats [26, 61, 62]. The relative abundance of the genus *Succiniclasticum* was improved by supplementation. This genus converts succinate to propionate [63]. Previous studies [64, 65] explained that cattle with higher propionate-producing rumen bacteria such as *Succiniclasticum* and *Prevotella* and lower abundance of genus *Mogibacterium* had lower methane emission and higher feed efficiency. This finding highlights the importance of phytogenic supplementation and explains the positive correlation between RGR, rumen fermentation parameters and *Prevotella*.

### Rumen archaea

Dietary intervention is the main determiner of rumen archaea by affecting hydrogen producers and utilizers that affect the available growth substrates for methanogens [59]. Consequently, variations in the composition and relative abundances of rumen methanogens due to tannin and herbal mixture supplementation are explained. The archaeal community was dominated by the genus *Methanobrevibacter* and *Candidatus Methanomethylophilus*, which agrees with the results on goats [66]. Previous studies [65, 67, 68] reported that phytochemicals such as tannin and flavonoids declined rumen methanogens and methane production and improved feed efficiency, which supports the decline in predicted methane production and *Methanobrevibacter* in the current study.

*Methanobrevibacter*, the main methane producer in the rumen, uses H_2_ besides other substrates to produce methane [69, 70]. Arndt et al. [71] reported that high-efficiency dairy cow exhibited lower methane emissions, and lower relative abundance of *Methanobrevibacter*, which highlights the benefits of phytogenic supplementation and explains the negative correlation between RGR, *Methanobrevibacter*, and methane production (Fig. 3). A similar conclusion was obtained by Zhou et al. [72] who explained that *Methanobrevibacter* uses acetate as a substrate for CH_4_ production, which leads to energy loss in the form of methane and decline in animal feed efficiency.

The possible explanations for *Methanobrevibacter* decline is the low hydrogen availability [70]. This speculation is supported by the increase of *Succiniclasticum* and *Prevotella*, which use hydrogen to produce propionate [64]. A similar conclusion was obtained by Hassan et al. [25] who explained that essential oils of *Origanum vulgare*, garlic, and peppermint increased the propionate-producing bacterial families such as Succinivibrioanceae, which decreases the available hydrogen for methanogenesis. Another explanation, phytochemicals have antimicrobial properties against rumen protozoa, the main hydrogen providers to methanogens [44]. A similar conclusion was obtained by Li et al. [23] on buffalo supplemented with flavonoides.

Previous studies [73, 74] reported that saponins improved animal performance by suppressing rumen methanogens and methane production. Aboagye and Beauchemin [75] and Tawab et al. [76] explained that phytochemicals disrupt the cell wall of methanogens causing toxicity. Moreover, Szulc et al. [77] indicated that polyphenols inhibit the populations and/or activity of methanogens by changing the rumen environment (pH value) and the toxic effect on methanogens. Furthermore, previous studies [25, 78] reported that the relative abundance of *Methanobrevibacter* was decreased by chestnut tannin supplementation and essential oils. *Candidatus Methanomethylophilus* is an H_2_-dependent methylotrophic methanogen and derives its energy from the metabolism of methanol and methylamine [79, 80]. Previous studies [81–83] indicated that higher relative abundance of *Candidatus Methanomethylophilus* was associated with improved feed efficiency, and average daily gain, and lower methane emission in sheep and steers, which supports the positive correlation between RGR, rumen fermentation parameters, and the relative abundance of *Candidatus Methanomethylophilus* (Fig. 3). These findings highlight the tannin and herbal mixture as suitable additives to improve feed efficiency and decreases methane emissions. The increase in the relative abundance of *Candidatus Methanomethylophilus* could be a result of the availability of methanol and methylamine in the rumen, which are produced via the fermentation of pectin by pectinolytic Lachnospiraceae and Spirochaetaceae [84]. Li et al. [85] reported that a higher abundance of *Candidatus Methanomethylophilus* was noted in the rumen of Sika Deer fed a high concentration of tannin-rich oak leaves. Choi et al. [86] reported that *Candidatus Methanomethylophilus* was enriched in the rumen of goats supplemented with *Pinus koraiensis* which is rich in essential oils.

### Histology of the rumen papillae

Previous studies [87, 88] indicated that dietary intervention affects the morphology of rumen papillae. The normal appearance of rumen papillae was noted in the control and herbal mixture-supplemented goat groups. A similar finding was observed by Petric et al. [21] on lambs supplemented by an herbal mixture. Tannin supplementation in the T group shortened the rumen papillae, which agrees with Hill [89] on Impala grazed on *Combretum imberbe* with high-tannin content [90]. This conclusion was confirmed by Brown et al. [91] who noted the atrophy of the rumen papillae in sheep-fed acacia. Redoy et al. [44] explained that increased VFA production in the rumen might increase the size of rumen papillae. Ghandour [92] found that tannins in *Acacia* hay had a negative effect on histological changes on rumen of sheep as there was a focal inflammatory cells infiltration and edemas in the lamina propria.

## Conclusion

Herbal mixture contains ginger, garlic, Artemisia, and turmeric improved the relative abundance of fiber-degrading bacteria, and decreased the major methane-producing archaea. Consequently, the rumen fermentation parameters were improved with balanced rumen pH and the morphology of rumen papillae was not affected negatively compared to tannin group (T). Therefore, it can be concluded that the herbal mixture in this study could be used as feed additive to alter rumen microbiota and improve rumen fermentation.

## Data Availability

The datasets generated and/or analyzed during the current study are available in SRA at https://www.ncbi.nlm.nih.gov/sra/PRJNA1008569.

## Abbreviations

ADF: Acid detergent fiber
NH3-N: Ammonia nitrogen
ASV: Amplicon Sequence Variants
CO2: Carbon dioxide
CT: Condensed tannins
CP: Crude Protein
CPI: Crude protein intake
DM: Dry matter
DMI: Dry matter intake
EE: Ether extract
EEI: Ether extract intake
FID: Flame ionization detector
HPLC: High-performance liquid chromatography
H2: Hydrogen
CH4: Methane
NDF: Neutral detergent fiber
NDFI: Neutral detergent fiber
OMI: Organic matter intake
PCA: Principal component analysis
PCoA: Principal Co-ordinate analysis
RGR: Relative Growth Rate
VFA: Volatile fatty acids
